# Resveratrol Ameliorates High Glucose and High-Fat/Sucrose Diet-Induced Vascular Hyperpermeability Involving Cav-1/eNOS Regulation

**DOI:** 10.1371/journal.pone.0113716

**Published:** 2014-11-24

**Authors:** Xiao lin Peng, Wei Qu, Lin zhi Wang, Bin qing Huang, Chen jiang Ying, Xiu fa Sun, Li ping Hao

**Affiliations:** 1 Department of Nutrition and Food Hygiene, School of Public Health, Tongji Medical College, Huazhong University of Science and Technology, Wuhan, PR China; 2 Nanshan Centre for Chronic Disease Control, Shenzhen, PR China; 3 Department of Hygiene, College of Basic Sciences, Binzhou Medical University, Yantai, PR China; 4 Hubei Key Laboratory of Food Nutrition and Safety, School of Public Health, Tongji Medical College, Huazhong University of Science and Technology, Wuhan, PR China; 5 Key Laboratory of Environment and Health, Ministry of Education and Ministry of Environmental Protection, and State Key Laboratory of Environmental Health (Incubating), School of Public Health, Tongji Medical College, Huazhong University of Science and Technology, Wuhan, PR China; Texas A&M University Health Science Center College of Medicine & Baylor Scott and White Health, United States of America

## Abstract

Vascular endothelial hyperpermeability is one of the manifestations of endothelial dysfunction. Resveratrol (Res) is considered to be beneficial in protecting endothelial function. However, currently, the exact protective effect and involved mechanisms of Res on endothelial dysfunction-hyperpermeability have not been completely clarified. The aim of present study is to investigate the effects of Res on amelioration of endothelial hyperpermeability and the role of caveolin-1 (Cav-1)/endothelial nitric oxide synthase (eNOS) pathway. Adult male Wistar rats were treated with a normal or high-fat/sucrose diet (HFS) with or without Res for 13 weeks. HFS and in vitro treatment with high glucose increased hyperpermeability in rat aorta, heart, liver and kidney and cultured bovine aortic endothelial cells (BAECs), respectively, which was attenuated by Res treatment. Application of Res reversed the changes in eNOS and Cav-1 expressions in aorta and heart of rats fed HFS and in BAECs incubated with high glucose. Res stimulated the formation of NO inhibited by high glucose in BAECs. Beta-Cyclodextrin (β-CD), caveolae inhibitor, showed the better beneficial effect than Res alone to up-regulate eNOS phosphorylative levels, while NG-Nitro-77 L-arginine methyl ester (L-NAME), eNOS inhibitor, had no effect on Cav-1 expression. Our studies suggested that HFS and in vitro treatment with high glucose caused endothelial hyperpermeability, which were ameliorated by Res at least involving Cav-1/eNOS regulation.

## Introduction

Vascular hyperpermeability is one of the manifestations of endothelial dysfunction [1]. Evidence demonstrated that endothelial dysfunction is recognized as initial step in the atherosclerotic process and is well advanced in diabetes [2]. Therefore, early intervention of vascular hyperpermeability is an important measure for prevention of diabetes and cardiovascular disease.

Resveratrol (Res; 3, 5, 4′-trihydroxystilbene) is a polyphenolic phytoalexin found in grapes, red wines and other products. For many decades, great interests to Res have been focused on its anti-carcinogenic, anti-inflammatory and cardioprotective properties [3].

Res is beneficial in combating against vascular protection of diabetes and cardiovascular disease, however, the mechanisms referred were complicated and confused. When adenosine monophosphate-activated protein kinase (AMPK) mediated reduction of superoxide level [4] or vascular endothelial growth factor (VEGF) pathway [5], [6] were frequently mentioned, caveolae/caveolin-1(Cav-1) attracts the interest of researchers [7], [8] because it is involved in the delivery of macromolecules across endothelium [9]–[11]. Caveolae are 50 to 100-nm cell-surface plasma membrane invaginations and abundant in endothelial cells. Cav-1 is the principal marker of caveolae. Recent studies show Cav-1 acted on vascular hyperpermeability by inhibiting endothelial nitric oxide synthase (eNOS) [12]. eNOS is an important signaling molecule in control of vascular permeability [13], and it's the major source of NO. Although eNOS and NO are considered as major player in the endothelial function, studies provided dubious results about the effect of Res on them. As shown as previously released data, long-term treatment of cultured endothelial cells with red wine polyphenols (RWP, especially Res) significantly enhance eNOS expression and subsequent NO release from endothelial cells against endothelial dysfunction [14], [15]. However, Napoli et al. found consumption of red wine (Res) failed to affect vascular reactivity and NO production in type 2 diabetic patients [16]. In addition, Cav-1 was reported to be involved in the anti-cancer activity of Res in a human hepatocellular carcinoma (HCC) model [17], while limited data existed regarding the modulation of Res on endothelial permeability through Cav-1 in vitro and in vivo.

Therefore, we aimed to evaluate the effects of Res on hyperpermeability induced in rats by high fat/sucrose diet (HFS) and in bovine artery endothelial cells (BAECs) exposed to high glucose from the viewpoints of vasoprotective properties and also explore the role of Cav-1/eNOS/NO pathway involved.

## Materials and Methods

### Materials

Res is provided by Biological Co., Ltd. NanJing, Zelang (JiangSu, China). β-Cyclodextrin (β-CD) and NG-Nitro-77 L-arginine methyl ester (L-NAME) were from Sigma-Aldrich Co. (USA). NO assay kit was from Nanjing Jiancheng biological company (China). Trizol Reagent was obtained from Invitrogen. Reagents for Real-Time PCR were gained from Takara. Reagents used for gel electrophoresis were from Santa Cruz (USA). Antibodies were obtained from the following sources: anti-Cav-1, anti-eNOS, anti-p-eNOS (Ser1177), anti-β-actin, anti-rabbit IgG (Santa Cruz, USA), HRP-labeled anti-goat IgG and HRP-labeled anti-mouse IgG (Sigma, USA).

### Cell culture

Bovine artery endothelial cells (BAECs, NO.C-003-5C) obtained from Health Science Research Resources Bank (Osaka, Japan) were maintained at 37°C in 5% CO_2_ in DMEM containing 10% FBS as described previously [18]. Confluent cultures were detached using trypsin/EDTA and plated on 96-well plate for evaluating cell viability, on 24-well millicell filters (Millipore, USA) for measuring endothelial permeability, and on 6-well plate for detecting NO production and mRNA expression or on 100-mm-diameter dishes for protein expression and phosphorylating signal analysis.

### Assessment of cell viability

Cells were seeded at a density of 1×10^4^ cells/well in 96-well plate. After reaching 90% confluence, the cells were treated with 5.5 mM or 33 mM Glu in the absence or presence of Res for 24 h., followed by measurement of cell viability according to the Cell Counting Kit-8 manufacturer's instructions (Dojindo, Japan). Briefly, the cultured wells were treated for 4 h with solution (10 µl/well) provided in the kits, and the level of cellular viability was measured at 450 nm by using a microplate reader.

### Measurement of endothelial permeability in BAECs

BAECs layer permeability was measured as describes previously [19], [20]. Briefly, BAECs were seeded to 0.4 µm 24-well millicell filters (Millipore. USA). After incubation in high Glu with or without Res for 24 h, FITC-dextran (1 mg/ml, 40 kDa, Molecular Probes) was added to the upper compartment of the filter inserts for 3 h before determination. Mannitol is commonly used as an osmotic control. The fluorescence in the medium collected from the lower chambers was evaluated with excitation at 419 nm and emission at 528 nm.

### NO measurement

After different treatments, NO contents in supernatants collected from cultured BAECs were detected using a NO assay kit (Nanjing. China). The cellular protein concentration was quantified with BIO-RAD DC protein assay reagent. Total nitrite concentration was expressed as µmol/L per milligram protein.

### Animals and Treatments

10 male Wistar rats (170±10 g) were given Res [50 mg/kg body weight (bw)] by intragastric with normal diet. The levels of trans-Res were detected in the plasma as previous method [21], [22] using high performance liquid chromatography (HPLC) method with fluorescence detection (FLD) to explore the bioavailability of Res and confirm the experimental concentrations of this study.

All 24 male Wistar rats (170±10 g) obtained from Sino-British Sippr/BK Lab Animal Ltd. (Shanghai, China) were maintained in constant temperature-controlled room (20–22°C) with controlled lighting (12 h light/dark cycles). The animals were cared for according to the Guiding Principles in the Care and Use of Animals. The experiments were approved by the Tongji Medical College Council on Animal Care Committee. Animals were randomly assigned to four groups of six animals each according to body weight and plasma glucose and fed one of the following diets ad libitum for 13 weeks: normal diet (CON) containing 12.96 KJ/g (63.3% of energy from carbohydrate and 10.2% of energy from fat), high-fat/sucrose diet (HFS) containing 17.09 KJ/g [27.4% of energy from carbohydrate (19.65% starch, 7.8% sucrose) and 54.2% of energy from fat], HFS plus Res1 [50 mg/kg body weight (bw)] and HFS plus Res2 [100 mg/kg body weight (bw)].

Energy intake was monitored every other day, and body weight was monitored weekly. Fasting blood was collected from the tail vein monthly. 13 weeks later, tissue permeability was estimated and blood samples were collected by decapitation. Parts of aorta and heart were stored at −80°C for Real Time PCR and Western Blotting analysis.

### Analysis of blood samples

Levels of fasting serum glucose were assayed with glucose oxidase-peroxidase assays (BIOSINO Biotechnology and Science Inc., Beijing, China). Levels of total cholesterol (TC) and triglycerides (TG) were measured by using enzymatic colorimetric assays (Nanjing Jiancheng Bioengineering Institute, Jiangsu, China) following the manufacturer's guidelines.

### Measurement of endothelial permeability in aorta and heart

Tissue permeability was quantified by measuring albumin leakage from aorta and heart blood vessels using the Evans blue albumin method. Briefly, Evans blue dye (Sigma) was dissolved in normal saline. At the end of the experiment, rats were anesthetized in a specimen jar contained cotton ball infiltrated with diethyl ether, and the dye (45 mg/ml) was injected quickly over 10 s through the caudal vein once rats were unresponsive and limply. The rats were sacrificed 2 h later to ensure complete dye circulation. Blood samples were collected, and thoracic aorta and heart were rapidly separated. One part of the tissues was incubated individually in formamide (Shanghai, China) for 24 h at 60°C to extract the Evans blue dye from the tissues, and the rests of aorta and heart were immediately frozen in liquid nitrogen, and then stored at −80°C until further tested. The extracts were centrifuged at 10,000× g for 45 min, and then the absorbance in 200 µl of the supernatant was measured at 620 nm.

### Real time PCR

Cav-1 and eNOS mRNA levels were quantified by real-time PCR method. Briefly, total RNA was extracted from aorta, heart and BAECs following the manufacturer's protocol, and converted to cDNA after quantification. Quantitative Real-time PCR was performed with the following parameters: 1 cycle, 95°C, 5 s; 40 cycles, 95°C 10 s, 57°C, 30 s, then added a dissociation curve. Changes of gene expression were determined by the Comparative Ct method. The mRNA of GAPDH was quantified as an endogenous control. The gene and primer information of rats were as follows: the forward and reverse primers for GAPDH were: GCA AGT TCA ACG GCA CAG and GCC AGT AGA CTC CAC GAC AT; for Cav-1 were: AAC AGG GCA ACA TCT ACA A and TCC GCA ATC ACA TCT TCA; for eNOS were: GGC ATC ACC AGG AAG AAG A and CAG AGC CAT ACA GGA TAG TCG. The gene and primer information of BAECs were as follows: the forward and reverse primers for GAPDH were: ATG ACC ACT GTC CAC GCC AT and GCC TGC TTC ACC ACC TTC TT; for Cav-1 were: TCA GCC GTG TCT ATT CC and ATT TCT TTC TGC GTG TTG; for eNOS were: CAA GCG AGT GAA AGC AA and ATC GCC ATT CCC AAA G.

### Western blotting

Equal amount of protein was separated by SDS-PAGE and transferred to PVDF membranes by electromembrane transfer. After blocking, membranes were incubated with primary antibodies under overnight at 4°C, followed by incubation with the secondary antibodies conjugated to horse-radish peroxidase. Bound antibody was detected with enhanced chemiluminescence's reagent (Millipore kit). Immunoreactive protein quantity was assessed by scanning densitometry.

### Statistical analysis

Data were expressed as Mean ± SD. Statistical significance was analyzed by ANOVA and multiple comparisons were done by LSD (least significant difference) t-test, and correlation analyses were also done used SPSS version 12.0 statistical software package (SN: 59245 46841 40655 89389 09859 21671 21957 29589 12). p<0.05 was considered different.

## Results

### Res prevented hyperpermeability in high Glu cultured BAECs

**Figure 1:**
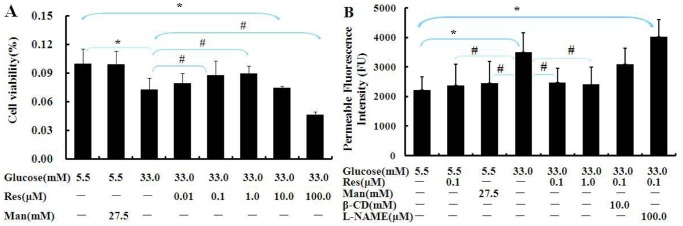
Res prevented hyperpermeability in high Glu-cultured BAECs. (**A**) Effect of Res on the viability of BAECs. (**B**) After pretreatment with β-CD or L-NAME for 1 h, BAECs were incubated with media containing 5.5 mM or 33 mM Glu in the absence or presence of Res for 24 h, and then added FITC-dextran for 3 h before determination. Man (mannitol, 27.5 mM) is an osmotic control. Data are Mean ± SD (n = 6). *: p<0.05 versus normal Glu; #: p<0.05 versus high Glu.

To ascertain the concentration range of Res on protection of BAECs against high Glu injury, we tested the effect of Res (0.01, 0.1, 1.0, 10.0 and 100.0 µM) on the viability of BAECs incubated with high Glu for 24 h with CCK-8 assay. It showed that Res (0.01, 0.1, 1.0 µM) played a protective effect on cell viability in a dose-dependent manner. BAECs exposed to high concentration of mannitol were used to determine whether or not the high Glu-induced change in viability was due to the change in osmolarity. BAECs viability did not decrease in mannitol group, suggesting the high Glu-reduced viability was not caused by increased osmolarity (**Fig. 1A**).

**Fig. 1B** showed high Glu significantly increased BAECs permeability, while the increase was prevented by Res (p<0.05). To further explore the mechanism by which Res ameliorates permeability, we used the eNOS inhibitor L-NAME and caveolae inhibitor β-CD. The preventive effect of Res on hyperpermeability was eliminated largely by L-NAME (eNOS inhibitor), but only a decrease appeared under the treatment of caveolae inhibitor, β-CD, and there was no significant differences.

### Res stimulated NO production

**Figure 2:**
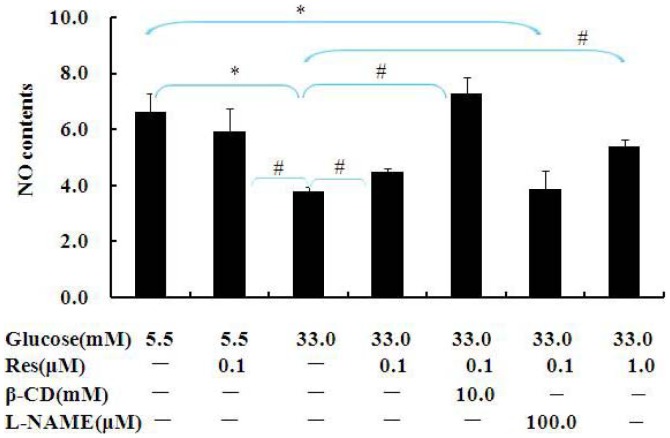
Res prevented decrease in NO production in BAECs. BAECs were pretreated with L-NAME or β-CD for 1 hour and then incubated with 5.5 mM or 33 mM Glu in the absence or presence of 0.1 µM Res for 24 h. The supernatants were collected for NO measurement. Values are shown as Mean ± SD (n = 3). *: p<0.05 versus Con; ^#^: p<0.05 versus high Glu.

To further evaluate a possible contribution of NO in the anti-hyperpermeability effect of Res, we performed an NO assay after BAECs treated with β-CD or L-NAME in the presence of 0.1 µM Res. We observed Res stimulated NO production inhibited by high Glu. However, the inhibitor of eNOS, L-NAME restricted NO increase induced by Res. It is obvious that β-CD which is an inhibitor of caveolae show the same beneficial effect as Res on NO contents (**Fig. 2**). Taken together, these results indicate that Res protects endothelial permeability through regulating NO production. Since NO-driven functions in endothelial cells were documented to be influenced by changes of Cav-1 and/or the interaction of eNOS with Cav-1, the following study was performed to clarify whether Res has an effect on the expressions of eNOS and Cav-1.

### Res reversed eNOS and Cav-1 expressions in high Glu cultured BAECs

**Figure 3:**
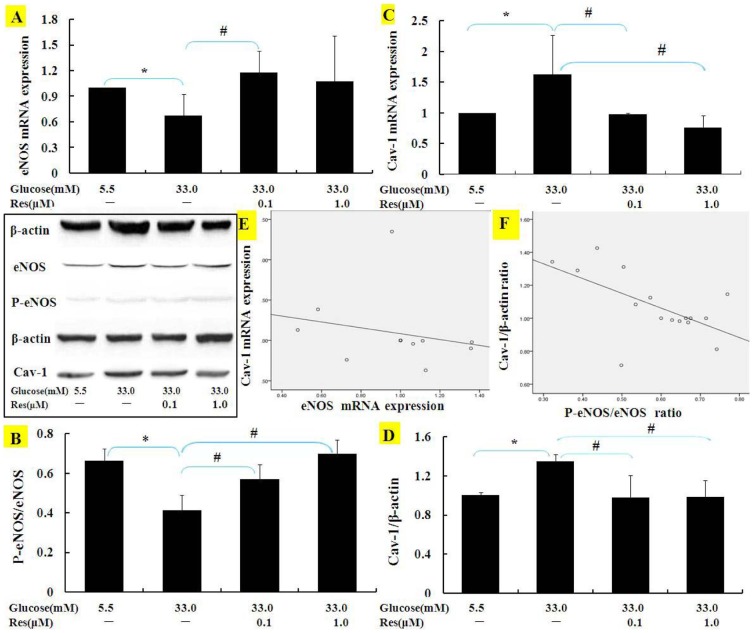
Effects of Res on eNOS phosphorylation and Cav-1 expressions in BAECs. The mRNAs and proteins of eNOS (**A, B**) and Cav-1 (**C, D**) in BAECs were detected by using real-time PCR or Western blotting BAECs were treated with Res under normal or high Glu condition for 24 h. Results in A, B, C, D were scatter-plotted and shown in **E and F**. Results represent Mean ± SD (n = 3). *: p<0.05 versus normal Glu; #: p<0.05 versus high Glu.

Endothelial NOS mRNA and phosphorylative levels in BAECs were enhanced by treatments with 0.1–1.0 µM Res (**Fig. 3A and 3B**). Meanwhile, Res obviously reduced Cav-1 mRNA and protein expression induced by high Glu (**Fig. 3C and 3D**).

The scatter-plotted of the levels of eNOS and Cav-1 showed in **Fig. 3E and 3F**, and the expressions of both the molecules were negatively correlated in culture BAECs.

### Cav-1/eNOS pathway may play an important role in Res ameliorating hyperpermeability

**Figure 4:**
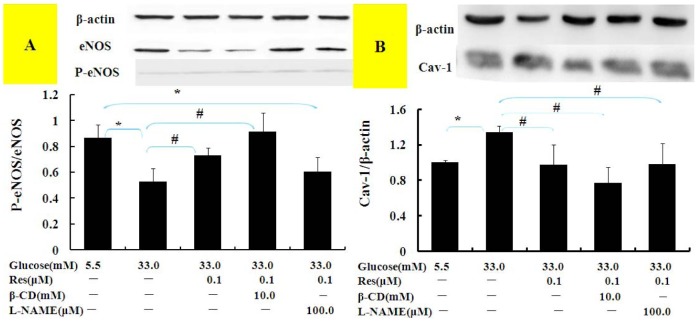
Influence of β-CD and L-NAME on eNOS phosphorylation and Cav-1 expression in BAECs. After pretreatment with β-CD and L-NAME for 1 h, BAECs were incubated with media containing 5.5 mM or 33.3 mM Glu with or without Res for 24 h. Cell lysates were immunoblotted with specific antibodies. Results represent Mean±SD of three independent experiments. *: p<0.05 versus Normal group; ^#^: p<0.05 versus high Glu.

To further confirm the role of Cav-1/eNOS pathway and to assess the relationship between Cav-1 and eNOS, we treated cell cultures with β-CD, an inhibitor of Cav-1, and with L-NAME, an inhibitor of eNOS. Results (**Fig. 4**) demonstrated that L-NAME inhibited eNOS phosphorylative levels increased by Res, and β-CD inhibited Cav-1 protein expression more than Res treatment alone. Simultaneously, β-CD showed the better beneficial effect than Res alone to up-regulate eNOS phosphorylative levels (p<0.05), while L-NAME had no effect on Cav-1 protein expression.

### Animal characterization

As shown in **Table 1**, rats fed with HFS gained more weight significantly than others since the sixth weekend, while Res treatment decreased the weight of rats. The total food consumption of HFS diet rats was significantly less than normal diet rats, however, there was no obvious difference in energy intake between CON and HFS groups, and Res inhibited the energy intake of rats (**Table 2**).

**Table 1:** Effect of Res on body weight (BW, g) in rats fed HFS diet.

Group	0^th^ week	3^th^ week	6^th^ week	9^th^ week	13^th^ week	BW gain
CON	195.3±12.0	300.9±20.3	338.4±22.3	378.1±21.9	385.3±31.5	190.0±29.2
HFS	194.3±13.5	300.9±13.0	361.0±20.6*	416.6±21.7*	453.7±22.9*	259.4±29.6*
HR1	200.3±8.1	289.0±11.8	343.1±10.4	390.3±19.0#	415.4±27.2#	215.1±22.3#
HR2	201.3±8.3	290.4±12.2	335.7±12.7#	391.7±19.4#	407.3±18.9#	206.0±18.4#

Data are Mean ± SD (n = 6).

*: p<0.05 versus CON;

# : p<0.05 versus HFS.

CON: normal diet-fed rats, HFS: high-fat/sucrose diet-fed rats, HR1: 50 mg/kg·bw Res+HFS, HR2:100 mg/kg·bw Res+HFS.

**Table 2:** Effect of Res on food consumption (FC, g) and energy intake (EN, KJ) of rats.

Group		3^th^ week	6^th^ week	9^th^ week	13^th^ week	Total
FC	CON	169.7±10.6	144.1±11.4	151.1±5.9	167.0±17.0	1958.8±86.3
	HFS	109.8±6.2*	113.1±5.4*	112.6±3.6*	121.2±4.0*	1469.1±63.0*
	HR1	100.4±3.3#	103.0±8.5#	101.5±6.2#	114.4±4.4	1319.8±52.5* #
	HR2	105.7±3.6	103.6±6.3	111.4±9.8	105.5±14.0#	1387.6±83.4*
EN	CON	2198.5±138.0	1867.6±148.2	1957.7±76.1	2163.4±219.9	25381.5±1117.9
	HFS	1877.6±105.8*	1933.8±92.9	1924.6±61.7	2072.0±68.1	25113.7±1076.6
	HR1	1742.9±83.3	1771.4±153.6#	1807.8±170.1	1992.9±73.2	23181.4±1411.0#
	HR2	1806.3±62.4	1770.7±108.1#	1905.3±168.5	1803.5±239.5#	23720.8±1425.5

Data are Mean ± SD (n = 6).

*: p<0.05 versus CON;

# : p<0.05 versus HFS.

CON: normal diet-fed rats, HFS: high-fat/sucrose diet-fed rats, HR1: 50 mg/kg·bw Res+HFS, HR2:100 mg/kg·bw Res+HFS.

HFS diet for 13 weeks caused an increase of serum glucose and serum lipid concentrations and this effect was attenuated with daily injection of Res (**Table 3**).

**Table 3:** Effect of Res on plasma glucose and lipid concentrationof high-fat/sucrose diet rats.

Group		0^th^ week	4^th^ week	9^th^ week	13^th^ week
FPG(mmol/L)	CON	5.39±0.67	5.51±0.80	5.83±0.47	5.90±0.48
	HFS	5.50±1.16	5.21±0.34	6.66±0.63*	7.04±1.52*
	HR1	5.49±1.17	5.26±0.42	6.40±0.48	6.09±0.56
	HR2	5.87±0.40	5.91±1.13	6.48±0.44*	5.90±0.55#
TC (mmol/L)	CON	0.97±0.10	1.04±0.16	1.11±0.23	1.21±0.26
	HFS	1.04±0.20	1.21±0.15*	1.23±0.09	1.55±0.29*
	HR1	1.04±0.12	1.06±0.09	1.16±0.12#	1.25±0.23
	HR2	0.98±0.20	1.08±0.02	1.17±0.13	1.19±0.34#
TG (mmol/L)	CON	0.83±0.30	0.95±0.52	1.09±0.35	1.15±0.20
	HFS	0.86±0.28	0.95±0.28	1.11±0.28	1.50±0.23*
	HR1	0.87±0.25	0.96±0.28	0.96±0.10	1.01±0.25#
	HR2	0.86±0.24	1.05±0.27	1.09±0.36	1.28±0.48

Data are Mean ± SD (n = 6).

*: p<0.05 versus CON;

# : p<0.05 versus HFS.

CON: normal diet-fed rats, HFS: high-fat/sucrose diet-fed rats, HR1: 50 mg/kg·bw Res+HFS, HR2:100 mg/kg·bw Res+HFS.

### Res prevented hyperpermeability in HFS diet rats

Given that Res (0.01, 0.1, 1.0 µM) played a protective effect on cell viability in a dose-dependent manner, and Res in the range concentrations mentioned above could prevent hyperpermeability in high Glu cultured BAECs. Then, what is the rational for the doses of Res used in vivo to gain the effective plasma concentrations? Therefore, under the guidance of the previous literatures and the in vitro experiments, we carry out the metabolic experiment with the dose of 50 mg/kg Res.

**Figure 5:**
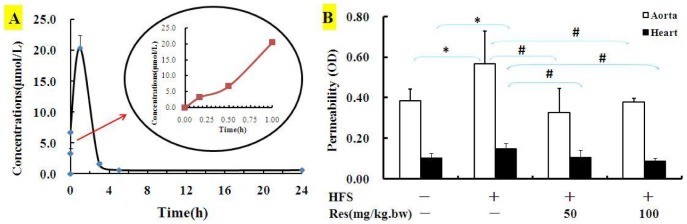
Res prevented hyperpermeability in high fat/sucrose diet rats. (**A**) Mean plasma concentration-time curves of resveratrol after the intragastric injections of 50 mg/kg.bw trans-Res to Wistar rats (n = 10). (**B**) Wistar rats were fed with either normal diet or HFS in the absence or presence of Res (50 and 100 mg/kg.bw) for 13 weeks (n = 6). Tissue's permeability was assessed by using Evan Blue dye. Data are Mean ± SD. *: p<0.05 versus CON; #: p<0.05 versus HFS.

The results showed that Res was quickly absorbed and reached its peak concentration (20 µM) approximately 1 h after the oral administration of 50 mg/kg Res to healthy rats, and then the plasma levels quickly declined to 1.6 µM in 3 h and then continued to decline to about 0.6 µM after Res intake for 5 h and lasted till 24 h (**Fig. 5A**), and the plasma concentrations were just consistent with the effective concentrations in vitro experiments.

Endothelial permeability in aorta and heart from HFS group increased significantly compared to CON group, while Res apparently attenuated the permeability in both tissues (**Fig. 5B**). It's interesting that the diet-induced permeability increase was minimal in other tissues (brain, lung, spleen, etc.) comparing with that of aorta and heart, and related data and involved mechanisms would be showed in next job.

### Res reversed eNOS and Cav-1 expressions in HFS diet rats

**Figure 6:**
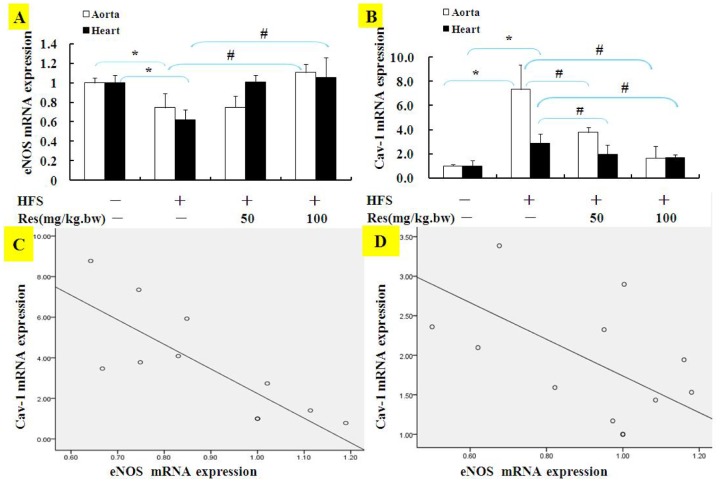
Effects of Res on eNOS and Cav-1 gene expressions in high fat/sucrose diet rats. The mRNAs of eNOS (**A**) and Cav-1 (**B**) in aorta and heart of rats were quantified with real-time PCR. Results in A, B were scatter-plotted and shown in **C and D**. Data are Mean ± SD (n = 3). *: p<0.05 versus CON; #: p<0.05 versus HFS.

HFS diet significantly decreased the mRNA expression of eNOS in aorta and heart of rats, while treatment with Res reverses the increase mentioned above (**Fig. 6A**). On the contrary, HFS increased mRNA expression of Cav-1 in aorta and heart tissues, while Res attenuated Cav-1 gene and protein expressions in **Fig. 6B**. As shown in the scatter-plotted in **Fig. 6C and 6D**, the expressions of the levels of eNOS and Cav-1 were consistently negatively correlated in both tissues of rats, especially in aorta, the correlation indexes was above 0.8, which was good correlation.

**Figure 7:**
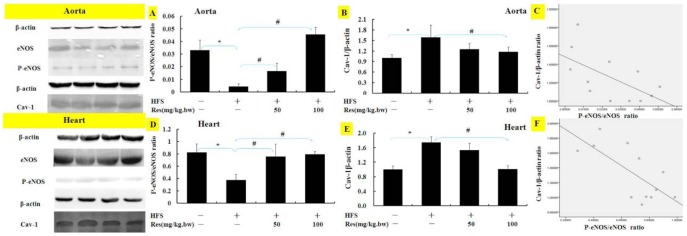
Effects of Res on eNOS phosphorylation and Cav-1 protein expressions in high fat/sucrose diet rats. The proteins of eNOS (**A, D**) and Cav-1 (**B, E**) in aorta and heart of rats were detected with Western blotting. Results in A, B, D and E were scatter-plotted and shown in **C and F**. Data are Mean ± SD (n = 3). *: p<0.05 versus CON; #: p<0.05 versus HFS.

Accordingly, phosphorylative protein expressions of eNOS in aorta and heart were obviously declining when fed HFS diet, while treatment with Res reverses the rise (**Fig. 7A and 7D**). As shown in **Fig. 7B and 7E**, HFS increased protein expression of Cav-1 in aorta and heart tissues, while Res attenuated Cav-1 protein expressions. The relevance of the results in **Fig. 7C and 7F** showed that Cav-1 was negative correlation with eNOS in aorta and heart tissues.

## Discussion

The principal findings of this study were as follows: (1) Diet high in fat/sucrose fed rats for 13 weeks or high Glu incubated BAECs for 24 h were able to cause vascular endothelial hyperpermeability; (2) Res markedly ameliorated endothelial permeability either HFS diet rats or BAECs in high Glu; (3) Res prevented endothelial injuries partly through augmentation NO contents by Cav-1/eNOS-dependent mechanisms. To our knowledge, we showed Cav-1/eNOS pathway takes part in the Res attenuating endothelial permeability in animals for the first time, and caveolae may be a new beneficial target of Res.

Epidemiological studies have demonstrated that the consumption of dietary polyphenol (Res etc.), such as moderate intake of red wine and polyphenolic-rich foods, i.e., fruits and vegetables, reduces the incidence of coronary heart disease and improves endothelial function [14], [23], [24]. Nevertheless, at present it's difficult to estimate the average dietary intake of Res in humans, and scarce information was available on biologically active dietary doses of Res. The dosages in our experiments were chosen guided by the previous literatures and preliminary experiments. Studies claimed the concentrations of daily dietary polyphenols (including Res) intake were about 10–500 mg/kg.bw [25]. Furthermore, researchers didn't observed adverse effect at 300 mg Res/(kg.bw·day) in rats [26]. Here 100 mg/kg.bw Res didn't show any harmful effect too, and Res supplementation had significant effects on neutralizing body weight, plasma glucose and lipid in rats of HFS diet. It's reported Res may exert regulatory effect on glycemic control via an improved glucose metabolism [27], activation of adenosine monophosphate-activated protein kinase (AMPK) [28] or Silent Mating Type Information Regulation 2 Sirtuins 1 (SIRT1) [29]. However, further researches are still needed to explore the exact mechanisms of hypoglycemic activity of Res.

Williamson et al. discovered vascular hyperpermeability appeared as early as 4 weeks in STZ-induced diabetic rats [30]. Here endothelial permeability significantly increased in the aorta and heart in HFS diet rats after 13 weeks, and the response was prevented by Res. It suggested Res was useful for preventing endothelial dysfunction associated with hyperglycemia or diabetes. Similarly in BAECs, high Glu-induced endothelial hyperpermeability was ameliorated by Res. Given that Res or its metabolites accumulate in vivo is still controversial, we investigated the bioavailability of Res to make sure the concentrations of Res applied in BAECs here were appropriate. The results showed that Res was quickly absorbed and reached its peak concentration (20 µM) approximately 1 h after the oral administration of 50 mg/kg Res to healthy rats, and then the plasma levels quickly declined to 1.6 µM in 3 h and then continued to decline to about 0.6 µM after Res intake for 5 h and lasted till 24 h. The concentration range of Res on BAECs in the present study was just based on the metabolic experiment. To be doubly sure, we also determined the effect of Res on the viability of BAECs, and found 0.01 µM to 1.0 µM Res were efficient, while 10.0 µM and 100.0 µM groups were observed an opposite effect. It's consistent with previous research [31], which considered 100 µM Res may exert harmful effects, however, some published studies revealed that 10 µM [32], [33] and 100 µM [34], [35] Res still showed beneficial effects in different models. We speculated it may be related to the kind of cell models and the ways of treatments, and the reason was worth further investigations.

It's proved that eNOS activity was affected by its gene expression and (reversible) post-translational modification of the enzyme [36], such as methylation of the eNOS promoter, NOS phosphorylation, and protein-protein interactions and so on. Here Res could increase eNOS phosphorylation levels in aorta and heart of rats. Aorta and heart were chosen to carry out further tests because they are rich in endothelial cells and most likely to be involved in CVD and certainly diabetes. Similar to our observation in tissues, Res up-regulated eNOS expression in BAECs in this work, which is consistent with other studies that eNOS plays an important role in the protective effect of Res [37], [38]. Meanwhile, we gained a deeper observation that Res seems to have no effect on preventing endothelial hyperpermeability, increasing NO production as well as eNOS phosphorylative expression when pretreated with eNOS inhibitor. Therefore, the findings suggested that Res may ameliorate endothelial hyperpermeability by inducing eNOS phosphorylation levels and NO production.

Although there is limited evidence Res prevented vascular endothelial against hyperpermeability directly via caveolae, researches showed that caveolae appear to play an indirect role in regulating vascular permeability via Cav-1 [12], [39]. Similarly in other studies [7], [40], we found rats fed with HFS diet and BAECs incubated with high Glu showed hyperpermeability and high expression of Cav-1 both in gene and protein level, when Res was applied here, Cav-1 expression was notably inhibited in vitro and vivo. Thus it can be seen that caveolae/Cav-1 participated in the protective effects of Res on endothelial dysfunction. Further data verified Cav-1 plays an inhibitory role in expression of eNOS and involves in the vasoprotective effects of Res when β-CD was applied and correlation analyses were done.

However, β-CD showed a weaker preventive effect than treatment of Res on hyperpermeability, which indicated Cav-1/eNOS may be not the only pathway of Res. Our former work found Res ameliorated hyperpermeability via VEGF/KDR pathway [41]. Additionally, investigations suggest that Res was one of the earliest identified energy-restriction mimetic agents (ERMAs) via SIRT1-dependent activation of AMPK [42], [43], and SIRT1 could activate eNOS and increase NO production and thus leads to endothelium-dependent vasodilation [44]. Moreover, the transcellular pathway in endothelial cells is dependent on caveolae trafficking, while Cav-1 is thought to participate both in transcellular and paracellular pathway to regulate endothelial permeability [45].

## Conclusions

This study provided a novel insight that Res plays anti-hyperpermeability by stimulating NO production via Cav-1/eNOS pathway. Then inhibiting “nitrosative stress” may be another pivotal way of the endothelium protective effect of Res. The job suggested that a right amount of Res intake may offer vascular protective effects in vivo and in vitro, and serve as a beneficial therapeutic supplement to prevent diabetic angiopathy. Though, of course, much more studies are needed, such as the role of paracellular pathway and AMPK/SIRT1 in the protective effect of Res, also the probable mechanisms involved the effects of Res on controlling the body weight and intake of energy, ameliorating blood glucose levels and lipid concentration.

## Supporting Information

Table S1 **Gene and primer information used for Real-time PCR.**
(DOC)
