# Supplementary material for: Resveratrol Ameliorates High Glucose and High-Fat/Sucrose Diet-Induced Vascular Hyperpermeability Involving Cav-1/eNOS Regulation
Source: PLoS One. 2014 Nov 24;9(11):e113716. doi: 10.1371/journal.pone.0113716 (PMC4242725; doi:10.1371/journal.pone.0113716)
Supplement: Table S1 — Gene and primer information used for Real-time PCR. (DOC) [file pone.0113716.s001.doc]

**Supporting Information**

**Table S1** Gene and primer information used for Real-time PCR

| **Gene** | **Accession number** | **Location** | **Source** | **primer** | |
| --- | --- | --- | --- | --- | --- |
|  |  |  |  | **sense** | **anti-sense** |
| Cav-1 | NM_174004.3 | chromosome 4 | bovine | 5'- TCAGCCGTGTCT ATTCC-3' | 5'-ATTTCTTTCTGCGTGTTG-3' |
|  | NM_031556.2 | 4q21 | rat | 5'-AACAGGGCAACA TCTACAA-3' | 5'-TCCGCAATCACATCTTCA -3' |
| eNOS | NM_181037.3 | chromosome 4 | bovine | 5'-CAAGCGAGTGAA AGCAA-3' | 5'-ATCGCCATTCCCAAAG -3' |
|  | NM_021838.2 | 4q11 | rat | 5'-GGCATCACCAGGAAGAAGA-3' | 5'-CAGAGCCATACAGGATAGTCG-3' |
| GAPDH | NM_001034034.2 | chromosome 5 | bovine | 5'-ATGACCACTGTCCACGCCAT-3' | 5'-GCCTGCTTCACCACCTTCTT-3' |
|  | NM_017008.4 | 4q42 | rat | 5'-GCAAGTTCAACGGCACAG-3' | 5'-GCCAGTAGACTCCACGACAT-3' |

Cav-1, caveolin-1; eNOS: endothelial nitric oxide synthase; GAPDH, glyceraldehydes-3-phosphate dehydrogenase.
